# A Statistical Approach to Detect Jamming Attacks in Wireless Sensor Networks

**DOI:** 10.3390/s18061691

**Published:** 2018-05-24

**Authors:** Opeyemi Osanaiye, Attahiru S. Alfa, Gerhard P. Hancke

**Affiliations:** 1 Department of Electrical, Electronic and Computer Engineering, University of Pretoria, Lynnwood Road, Pretoria 0002, South Africa; attahiru.alfa@umanitoba.ca (A.S.A.); gerhard.hancke@up.ac.za (G.P.H.); 2 Department of Electrical and Computer Engineering, University of Manitoba, Winnipeg, MB R3T 2N2, Canada

**Keywords:** wireless sensor networks, jamming attack, exponentially weighted moving average, inter-arrival time

## Abstract

Wireless Sensor Networks (WSNs), in recent times, have become one of the most promising network solutions with a wide variety of applications in the areas of agriculture, environment, healthcare and the military. Notwithstanding these promising applications, sensor nodes in WSNs are vulnerable to different security attacks due to their deployment in hostile and unattended areas and their resource constraints. One of such attacks is the DoS jamming attack that interferes and disrupts the normal functions of sensor nodes in a WSN by emitting radio frequency signals to jam legitimate signals to cause a denial of service. In this work we propose a step-wise approach using a statistical process control technique to detect these attacks. We deploy an exponentially weighted moving average (EWMA) to detect anomalous changes in the intensity of a jamming attack event by using the packet inter-arrival feature of the received packets from the sensor nodes. Results obtained from a trace-driven simulation show that the proposed solution can efficiently and accurately detect jamming attacks in WSNs with little or no overhead.

## 1. Introduction

WSNs in recent times have expanded their range of applications from their initial deployment for battlefield intelligence surveillance to areas such as emergency response support, meteorological weather forecasting, security applications and factory automation, just to mention a few. WSNs consiss of small and inexpensive sensor nodes without an existing infrastructure. They are often used to sense, process, transmit and receive information from the area they are deployed before it is conveyed to a base station. A typical WSN consists of hundreds to thousands of sensor nodes which can be categorized according to their structure (topology) and the environment in which they are deployed. Structurally, WSNs can be categorized according to the placement of the sensor nodes in the deployed environment [1]. These nodes can be of equal capacity, while others have varying capacity, depending on the architecture. The three main types of WSN structure are flat-based (tree), cluster-based and hierarchical [2]. Furthermore, the environments where the sensor nodes are deployed in a WSN can be grouped into five classes, namely: underground WSNs, terrestrial WSNs, underwater WSNs, multi-media WSNs and mobile WSNs [3].

The sensor nodes in a WSN are often deployed in remote, harsh and inaccessible areas and are often characterized by their resource constraints such as limited power, limited storage, limited bandwidth and short communication range. These, coupled with the vulnerability of the wireless medium (i.e., open and shared) have made sensor nodes susceptible to different security attacks such as the denial of service (DoS). A recent case of DoS attack on DYN, a major DNS provider, was reported on the 21st of October 2016 [4]. The Dyn DDoS attack was orchestrated by a botnet known as Mirai malware, which infected around 100,000 malicious terminal nodes and were mostly internet of things (IoT) devices. Experts have identified this distributed form of DoS as the most catastrophic DoS attack so far, with a magnitude in the range of 1.2 Tbps.

Jamming attacks are a form of DoS attack where an adversary transmits a high-range signal to disrupt communication. Generally, jamming can occur unintentionally in a wireless medium through situations such as noise, interference and collision [5], however, a jamming attack in a WSN is a deliberate attempt by an adversary to interfere with the physical transmission of signals during a communication process. The main aim of a DoS attack is to direct malicious signals towards the sensor nodes’ communication channels to deplete their resources such as the battery life, bandwidth, and storage in order to prevent transmitted sensor data from reaching its destination, thereby affecting its long-term availability [6]. A jamming attack in a WSN is catastrophic as it does not require any special hardware device or software to be perpetrated [5]. It can be carried out by passively listening to the wireless medium in order to broadcast on the same frequency band as that of the legitimate transmitting signal. A typical jamming attack is characterized by high energy efficiency, low detection probability, and anti-jamming resistance [7].

In WSNs, the physical layer and the MAC layer are the common targets of DoS jamming attacks. In the physical layer jamming attack, an adversary with a high transmission power signal can jam the communication medium, as most WSN deployments operate on a single frequency. Most traditional defence techniques rely on using a spread spectrum to mitigate physical layer jamming attacks. This technique is resource intensive and does not directly fit in WSNs due to the energy constraints of the sensor nodes. The access layer jamming attack, on the other hand, is perpetrated by either corrupting the control packets or deliberately reserving the communication channel for a maximum allowable number of slots, to ensure other nodes experience a lower throughput as a result of not being able to access the communication channel. Cases of jamming attacks at the network and transport layer have also been reported [5], where malicious packets are injected on certain routes and SNY flooding attacks are directed towards the sensor nodes to consume their resources.

Detecting jamming attacks in WSNs has been an ongoing research trend over the past decade as commonly proposed methods rely on dedicated devices or algorithms imbedded within the sensor nodes. These methods often use information obtained a priori about the communication behaviour during normal and jammed condition, which can be tracked using indicators and metrics obtained from different layers [8]. Example of these metrics are the received signal strength obtained at the physical layer and the packet delivery ratio at the application layer. Recently deployed methods have proposed a cross-layer architecture [9] to ease the collection of jamming attack indicators while other proposed methods have combined two or more metrics [10] to significantly improve the detection of jamming attacks.

Closely related works have proposed packet inter-arrival time as a metric in WSN to detect jamming attack situations. Wispernet, an anti-jamming technique for WSNs, was proposed in [11] to prevent jammers from predicting the length of activity on the channel and epoch. This ensures that the useful packet inter-arrival distribution cannot be determined and used to pre-empt an attack. Packet inter-arrival time has also been used in [12] to determine the most appropriate distribution at the cluster heads and relay nodes. Additionally, EWMA has also been proposed in the literature to detect the abnormal network traffic during the case of DoS attacks. An adaptive EWMA which uses an adaptive weighing function has been proposed in [13], as opposed to the constant weighting EWMA algorithm, to smoothen accidental errors to retain the exceptional mutation. Threshold-based EWMA has been proposed in [14] for intrusion detection based on their intensity.

Most of the past works have used two or more metrics to determine the presence of jamming attacks in WSNs and have also adopted a cooperative/distributed defence deployment approach by deploying the detection system on the ordinary member nodes, cluster heads and base station. This tends to add an extra overhead on an already resource constrained sensor node. Therefore, in this work, we harness the potential of EWMA, a statistical process control technique, to detect the mean shifts in event intensity during jamming attack in WSNs using the packet inter-arrival time (IAT). EWMA monitors and compares the observed packet sequence features against a normal profile obtained a priori to detect when a change has occurred. The choice of EWMA as opposed to other change-point methods is because EWMA combines both current and historical data to detect small and significant changes in time-series that can be detected easily and promptly. More importantly, the deployment of EWMA using packet IAT is lightweight, therefore it is perfectly suited to sensor nodes in WSNs that are characterised by energy constraints. The main contributions of this work are as follows:

- We present a novel jamming detection scheme by proposing EWMA for auto correlative data that observes the packet IAT which depends on the values that appear prior to or later in a series.
- We propose IAT as the sole metric to detect jamming attacks in WSN which reduces the complexity by offering a lightweight technique.
- Our proposed technique is a stepwise approach and deployed on the cluster head to detect jamming attacks in the member nodes and in the base station to detect jamming attacks in the cluster heads.

The rest of the paper is organised as follows: in Section 2, we present related work while in Section 3, we describe different jamming attack models in WSNs. Section 4 highlights the different possible metrics for detecting jamming attacks in WSNs while in Section 5, we present our proposed application of EWMA to detect DoS jamming attacks in WSNs. The validation of the proposed framework is presented in Section 6 while Section 7 concludes the paper.

## 2. Related Work

With the widespread deployment of WSNs, jamming attacks that send malicious radio signals to disrupt legitimate communication and consume resources, and its countermeasures have been studied in the literature. This is necessary as sensor nodes are vulnerable to this type of attack due to their architecture, hostile deployment and insecure routing protocols.

Mistra et al. [15] propose a centralised approach, a fuzzy inference-based system, to detect jamming attacks in the base stations by using three inputs received from each sensor node in the network. These inputs are the total packets received during a specific period, the number of dropped packets during that period and the received signal strength (RSS). The base station computes the power received during the jamming attack to find any difference in value between the current RSS and the normal RSS. These values are used by the base station to compute the packet drop per terminal (PDPT) and signal-to-noise ratio (SNR) which is further used as inputs for the fuzzy inference system to obtain the jamming index. The jamming index varies from 0 to 100 and is used to determine the intensity of the jamming attack, which can range between a situation of ‘no jamming’ to absolute jamming’. Strasser et al. [16] in their work identified the cause of bit errors for individual packets by analysing the RSS during the reception of these bits to detect reactive jamming attacks in sensor networks. This detection technique was based on predetermined knowledge, limited node wiring and error correction codes. Experimental results on Chipcon radios show its efficiency in detecting complex reactive jamming attacks without introducing additional overhead. In an attempt to detect complex reactive jamming attacks, Spuhler et al. [17] proposed a technique that estimates the probability of packet delivery during the synchronization stage of packet transmission. This technique ensures that jammers that target the physical layer of the WSN are detected by predicting the packet delivery probability using the chip error rate in the received preamble symbols.

A distributed approach to detect multichannel jamming attacks in WSN has been proposed by Guan and Ge [18]. In their approach, a piecewise homogenous Markov chain consisting of a complex two-level switching multichannel jamming attack model was developed by using multiple measurement channels to detect random attacks. Each state space of the Markov chain corresponds to the possible jamming attack modes. At a high level, the variations in probability of transition probability can be categorized into two, namely; stochastic switching and deterministic average dwell time switching. Cordero and Lisser [19] in their work analysed a heterogenous WSN under jamming attack using a two-player non-cooperative game chance constrained problem. The utility function used here is based on both signal interference and noise ratio at the receiver and a second order cone programme has been used to solve the game problem. Numerical results obtained show that the communication distance between the network elements must be put into consideration during the design of a detection technique for jamming attack.

Mpitziopoulos and Gavalas [20] propose a prototype node, Ares, which is a hybrid of frequency hopping spread spectrum (FHSS) and direct sequence spread spectrum (DSSS) to defend against jamming attacks in WSNs. In the proposed technique, the FHSS is used in the 5 GHz band with 51 frequency channels to generate a channel sequence using a key known only by the sensor nodes and the sink node. Each of these channel use DSSS modulation with 16-bits pseudo noise code which are derived from the same key used during FHSS channel generation. Simulation results show that Ares nodes can ensure satisfactory packet delivery rate with reduced energy requirements in a jammed WSN environment, as opposed to ordinary sensor network schemes.

Alnifie and Simon [21] proposed MULti-channel Exfiltration PROtocol (MULEPRO), a fully distributed protocol that is designed to evacuate data from jammed areas in response to a radio jamming DoS attack in a WSN. It functions by dynamically assigning nodes to different channels in the jammed area to defeat the attacker. The resilience of WSN routing protocols, such as AODV, DSR and the recent MPH to reactive jamming attack has been studied in [22]. The authors, thereafter proposed a modified version of the protocols, AODV-M, DSR-M and MPH-M that ensure that whenever the nodes running the detection algorithm detect an attack, the node is isolated and the routing protocols adapt their paths to avoid the isolated nodes.

A query-based jamming detection algorithm (QUJDA) has been proposed in [23]. QUJDA is an anomaly-based technique and its deployment is distributed. It functions by first differentiating between cases of attack condition and normal network condition using metrics such as bad packet ratio, packet delivery ratio and amount of energy consumed. QUJDA also ensures that sensor nodes relate with their neighbours to achieve a higher detection rate. A game theory approach has been proposed in [24] to ensure a high network lifetime and packet delivery ratio during multiple or single jammer attack in WSN using single-leader multiple-followers Stackelberg game theory. This approach, TC-JAM, uses a topological control technique, where the sink node acts as a leader to track nodes that have been affected by jamming attack. The sensor nodes, which acts as the followers, ensure an optimum transmission power level is achieved while making sure a large number of neighbour nodes are covered.

Many of these proposed techniques have used metrics from different layers to detect the presence of a jamming attack, however, there exist some network impairments such as collisions and packet failures due to interference and weak links that exhibit seemingly similar behaviour to a jamming attack. Therefore, some of these metrics that have been used in the literature to detect jamming attacks will produce high false alarm rates. In this work, we propose the EWMA algorithm and use the packet IAT as the sole metric to distinguish between normal traffic patterns and jamming attacks. EWMA is a statistical monitoring process technique that averages data and continually increases the weight of more recent values of the average variable. The packet IAT allows us to detect different forms of DoS jamming attacks in WSNs. To the best of our knowledge, this work is the first to use EWMA based on packet IAT to detect different forms of jamming attacks in WSNs.

## 3. Jamming Attack Models in WSN

Jamming can be described as any disruption or interference with the physical transmission and reception of wireless signals. This can either be intentional, in the form of radio frequency interference, unintentional in cases of collision and noise interference at the receiver, or in the context of an attack [5]. During a jamming attack, the jammers aim is to disrupt communication between the transmitter and receiver using minimal power. This attack is catastrophic as the jammer exploits the open and shared nature of the wireless medium to disrupt communications by reducing the signal to noise ratio (SNR). An attacker with enormous resources can continually jam the spectrum band to disrupt communication in the band. Additionally, an attacker can decide to jam the band intermittently, thereby forcing the receiving node to drop packets due to alteration [25]. The jamming device often used to perpetrate this attack selects a common channel that is currently been occupied by the nodes to block data from being successfully transmitted. The main aim of the jamming device is to occupy the channel and ensure that network is not available for legitimate nodes, while these nodes, on the other hand, attempts to maximize the use of the network.

One of the key components in determining a successful jamming attack is the SNR, defined as $P_{signal}/P_{noise}$, where *P* is the average power. The noise here can be described as an undesirable fluctuation of the electromagnetic spectrum from the antenna. A jamming attack is deemed successful if the SNR < 1. Two types of jamming attack have been described in [26], namely physical and virtual jamming. Practical examples of physical jamming attacks are radio jamming and collision attacks while virtual jamming attack consists of network allocation vector attacks and Request-To-Send/Clear-To-Send attack. Khatua et al. [27] in their work analysed the effects of jamming attacks on underwater sensor networks, while a further study into the effect of controllable jamming attacks on WSNs was presented in [5]. Mobile jamming attacks that can dynamically jam the critical path of WSNs have also been discussed in [28] while an energy efficient periodic jamming that attacks when the nodes are in listening state and sleeps at other times has been described in [15]. From the literature, four common jamming strategies have been identified, namely: constant jammer, deceptive jammer, reactive and random jammer [29,30]. Here, we briefly discuss each of these strategies:

*Constant jammer*: In a constant jammer attack, the jamming devices do not follow the laid down protocol before continually transmitting series of radio signals, electromagnetic waves or radio sequence of bits to interfere with legitimate transmitted signals in the network. The consequence of continually transmitting these malicious signals is that these random bits occupy the transmission channel of the network to starve transmissions initiated by legitimate nodes. Furthermore, constant jammer attacks can cause interference at the transmitting nodes to corrupt the signals received by the receiving nodes. One of the main disadvantages of constant jammer attack is the enormous energy consumption which drains the battery life of the node due to the continuous emission of signals. Constant jammers, therefore require a high amount of power to carry out this attack.

*Deceptive jammer*: Just as in the case of the constant jamming attack, a deceptive jammer continuously injects regular radio sequence of bits into the communication channel without gaps in between. However, different from a continuous jammer, it does not emit random bits but legitimate bit sequences. This often leads the network to believe that a normal transmission is taking place by legitimate nodes, thereby causing the legitimate nodes to wait indiscriminately in the listening state. Supposing a legitimate node has signals to transmit, it cannot change from listening state to send state because of the presence of a constant stream of signals in the channel. Deceptive jammers are difficult to detect and tend to be more effective when compared with constant jammers due to their impersonation features that makes them look like a legitimate transmission.

*Random jammer*: Random jammer attacks differ from both constant and deceptive attacks as they conserve their energy by alternating between jamming and sleep states. During the jamming process, the attacking node jams for a predetermined time before turning off its radio and switching to sleep mode. After a while, it reactivates the jamming process from the sleep mode and continually follows that sequence. During the jamming mode, it exhibits either the constant or deceptive jamming feature, while during the sleep mode, it does not dissipate energy, therefore reducing its rate of power consumption.

*Reactive jammer*: All the three previous jamming strategies discussed are active jammers, as they attempt to block the communication channel, regardless of the traffic pattern. An alternative to active jammers is the reactive jammer that presents a smarter and more power efficient approach [7]. Reactive jammers function by continually sensing the communication channel to detect when signals are being transmitted. On detecting a signal transmission in the channel, they start to transmit radio signals to cause collisions. Reactive jammers minimize the rate of power consumption and are very difficult to detect. The power they use when listening to a channel is less than that needed for jamming.

Having described the four common jamming strategies in WSNs, it has been observed that the different forms of DoS jamming attacks are automated and follow a seemingly similar and predictable pattern with regards to the packet IAT. This can therefore be tracked using a statistical process control, EWMA, to detect the mean shifts in the jamming event intensity. In this work, we focus mainly on reactive, constant and periodic jamming attacks in WSNs.

## 4. Detection Metrics for Jamming Attack

To detect the different forms of jamming attacks that has been described above, the metrics and indicators to achieve this must be defined. These metrics will therefore be closely monitored and captured during a normal traffic flow to detect the malicious node during a jamming attack. Common among these metrics are packet delivery ratio (PDR), packet sending ratio (PSR), bad packet ratio (BPR), bit error rate (BER), energy consumption amount (ECA), signal-to-noise ratio (SNR) [15] and packet IAT.

*Packet Delivery Ratio (PDR)*: PDR is the ratio of the number of packets that has been successfully delivered and acknowledged by the destination node to the number of packets sent by the transmitting node. The transmitting node only confirms the successful delivery of packets upon receiving an acknowledgment (ACK) packet from the destination node. This process involves a 4-way handshaking (RTS/CTS/DATA/ACK) where PDR is determined by comparing the RTS/DATA packets transmitted with the CTS/ACK packet received [23]. Mistra et al. [15] in their work described two types of PDR; the one obtained at the transmitter (source) and the other obtained at the receiver (sink). The former can only be possible if the network follows a reliable protocol like the TCP, where acknowledgment exist for every successfully delivered packet. PDR is a good metric to detect the different forms of jamming attacks at the MAC layer without much computational overhead [15], however when it involves TCP, where acknowledgment of packet is involved, PDR might not be appropriate for resource constrained sensor nodes.

*Packet Sending Ratio (PSR)*: PSR is the ratio of the number of packets that has been sent during a given time to the number of packets intended to be sent by the transmitting node during that given period. The number of packets intended to be sent can be obtained during a given period by first determining the time of the channel availability to the node at that given period and multiplying it by the transmission rate [15]. PSR can be used to determine how effective the jamming attack is on a transmitter using carrier sensing as its medium access policy. A typical scenario where PSR metric can be used to quantify jamming attack is in a situation where jamming signals render the medium busy as a result of carrier sensing which leads to transmission queue, therefore forcing the newly received packets on the full queue to be dropped [7].

*Bad Packet Ratio (BPR)*: BPR is the ratio of the number of damaged packets received by a node to the total number of packets received in a given period. The sensor nodes often determine this by using cyclic redundancy check (CRC) to check for damaged packets [23]. Any damaged packet is dropped and a negative result is returned while the good packets are received or queued for transmission. BPR has proven to be a very effective metric to detect different forms of jamming attack due to its ease to calculate. It can be easily adapted to resource constrained WSN environments where acknowledgement is not required.

*Bit Error Rate (BER)*: BER in WSN can be determined by computing the ratio of the number of corrupt bits to the entire bits received during a transmission session by a node. BER can be effective in detecting reactive jamming attacks, however, calculating BER can be very tasking, especially in situations where the nodes have to keep track of the BER for the entire radio links that are one-hop away [15].

*Energy Consumption Amount (ECA)*: ECA is the measure of the approximate amount of energy consumed by a sensor node over a period of time. This can be obtained by measuring the voltage drop of the battery component of a sensor node and multiplying its squared value with the time duration before finally dividing the result with the average electrical load of the node [15]. Adopting ECA as a metric to detect jamming attacks comes with its own issues, such as the difficulty in sampling the threshold energy consumption under different traffic load and the difficulty to detect jamming attack when the jammer uses a simple power attack.

*Signal-to-Noise Ratio (SNR)*: SNR can be obtained by finding the ratio of the received signal power to the received noise power at the node. It is a very effective metric to determine jamming attacks in the physical layer because for jamming to occur here, there must be a drop in the SNR value.

*Packet inter-arrival time (IAT)*: Packet IAT is the time that elapse between the receipt of a packet and subsequent packets in WSN [31]. The distribution of the packet IAT can be used to determine the probability of the occurrence of DoS jamming attacks during the transmission of signals.

In this work, we have used packet IAT as the sole metric to distinguish between normal traffic pattern and jamming attack. We believe that the IAT between packets of normal flow exhibit a strong regularity, thus can be used to track the presence of any form of jamming attacks which causes a mean shift due to the intensity of the jamming attack. Furthermore, the choice of only IAT is well suited to resource constrained sensor nodes, therefore using packet IAT presents a metric that can be easily measured by the node without introducing complexity and additional overhead to the system.

## 5. EWMA Algorithm

Jamming attacks in WSNs are perpetrated by malicious nodes in the network, with the aim to disrupt or interfere with the transmission and receipt of legitimate wireless signals among sensor nodes. Jamming attack affect the statistical features (for instance mean and variance) of a packet flow with temporal fluctuations. Statistical process control (SPC), can therefore be used to detect this anomaly by observing series of statistically homogeneous events.

SPC has been used over the years for monitoring processes and controlling the quality of manufacturing processes. SPC techniques can detect changes in the process mean, process variance and the relationship between multiple variables, which can be either univariate or multivariate [32]. CUSUM control charts, Shewhart control charts and EWMA control charts are examples of univariate SPC techniques which are often used in detecting shifts in mean values.

EWMA, which was proposed by Roberts [33], is an efficient statistical technique used in detecting small shifts in time-series data. It functions by first defining a threshold that delimits a standard behaviour before periodically handling updates on average of the observed data traffic [34]. EWMA is also characterised with its low complexity because the weighted average only needs to be updated for each newly observed data. The advantage EWMA has over other SPC techniques is that it combines current and historical data in a way that small shifts in time-series can be detected easily and quickly. On the other hand, other control charts such as Shewhart chart only consider the most current observations while neglecting the historical data [35]. EWMA use a weighing constant, lambda (λ), to determine the importance of both current and historical observations and to determine its sensitivity to small or gradual process drift. EWMA can be computed using [14]:

(1) z(t)=λ·x(t)+(1−λ)·z(t−1)                 t=1, 2, 3…n

where: z(t) is the mean of the historical data, x(t) is the observation at time *t*, *n* represents the number of observations to be monitored including z(0) and *λ* is the smoothing constant (0<λ≤1) that determines the depth of the EWMA.

The value of *λ* is used to determine how well the older data influences the calculation of the EWMA statistics. For instance, when *λ* has a value of 1, this means that only recent measurements influence the EWMA. Therefore, a large value of *λ* = 1 appends more weight to recent observations and older observations get less weight, while a small value of *λ* gives more weight to older observations [14]. Several values for *λ* have been suggested by previous authors, however the value of *λ* is not only dependent on the size of the mean shift, but also on the in-control Average Run Length [14]. Most often, a value less than 0.5 is taken for *λ*, however, using a very small value of *λ* might result in the algorithm being insensitive to attacks characterized by moderate intensity or having small duration. Therefore, in practice, *λ* is usually between 0.2 and 0.5.

The estimated variance of the EWMA statistic can be approximated using:

(2) $$\sigma_{z}^{2}=\sigma_{x}^{2}\cdot \left(\frac{\lambda }{2-\lambda }\right)$$

where *σ* is the standard deviation obtained from the historical data.

The control chart centre line is the target value, therefore the upper control limit (UCL) and lower control limits (LCL) can be determined using the equation below:

(3) $$UCL_{z}=z_{0}+f\cdot \sigma_{z}\;\;\;\;\;\;\;\;\;\;\;\;LCL_{z}=z_{0}-f\cdot \sigma_{z}$$

where the factor f is set to be equal to 3-sigma control limits.

The aim of this work is to determine the allowed EWMA values of the traffic in WSN which when exceeded are considered as a statistical anomaly thus signifying the presence of a jamming attack. During the detection of this attack, the main interest is to view the situation where the upper limit is exceeded. There are also instances where the observed features of the traffic falls below the lower control limit, which can also be as a result of network anomaly. In this work, we have developed an algorithm to detect jamming attacks using EWMA to observe the auto-correlated data, which is the packet IAT of the network traffic.

### Detection System Design

**Figure 1:**
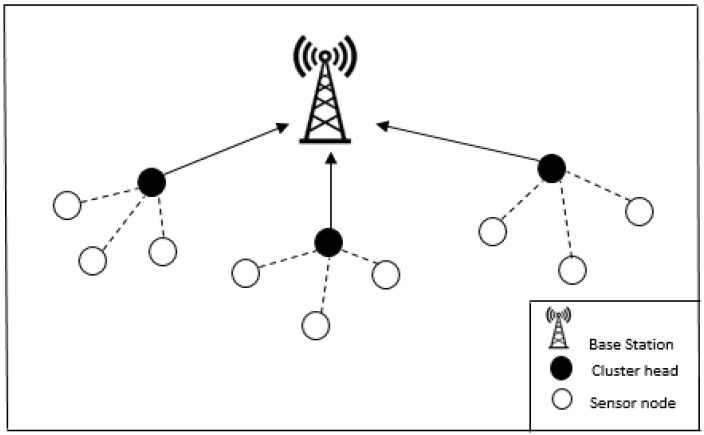
Cluster-based WSN topology.

In hierarchical WSNs, the sensing and monitoring function of the sensor nodes is distributed into different levels. Cluster-based WSNs are a typical example of hierarchical WSN, therefore in this work, we limit our scope to cluster-based WSNs. Deployment of sensor nodes into clusters has proved to be very efficient during sensing and monitoring, as it offers advantages such as fault tolerance, efficient data aggregation and reduced energy consumption. The architecture of the clustered WSN comprises of three types of nodes; namely, the member nodes, the cluster head and the base station (see Figure 1).

The member nodes forward sensed messages to their respective cluster heads through a process referred to as intra-cluster communication. The cluster head in turn aggregates and compresses the messages received from its members for upward transmission to the base station. Therefore, the architecture of cluster-based WSN can be regarded as a two-layer hierarchy WSN, where the cluster heads belong to the upper layer and the member nodes operate at the lower layer. The cluster heads in most cases perform more functions than the ordinary member nodes, therefore, cluster heads are often of higher capacity with respect to radio subsystem, processing subsystem, sensing unit and power supply. In this work, we assume that the cluster heads are of higher capacity, therefore our detection takes place in both the cluster heads and the base station (see Figure 1). All member nodes are within communication range and are one-hop away from the cluster head; likewise all the cluster heads and base stations. Our design approach is a stepwise decentralised approach, where jamming detection in member nodes are done by the cluster heads based on the input metric (i.e., IAT) received from member nodes. Just as in the case of cluster heads detection, the base stations in turn detect jamming attacks in the cluster heads based on the packet IAT input data received. As mentioned, both the cluster heads and the base station takes the packet IAT as input and use the EWMA algorithm to detect mean shifts in event intensity during jamming attack at each level in WSN.

**Figure 2:**
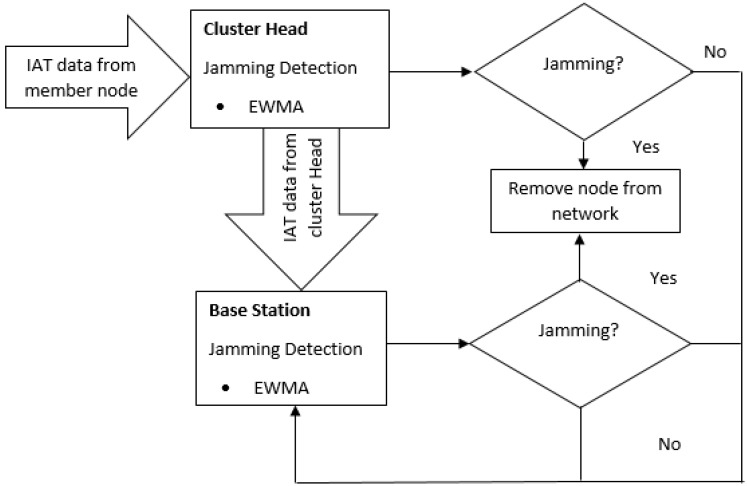
EWMA DoS jamming detection framework in WSN.

Figure 2 below depicts our DoS jamming detection framework in WSN. The member nodes sense information of interest in its deployed environment and use their inbuilt microcontrollers to process the sensed information. The corresponding result is sent to the cluster head that has the jamming detector. This detector measures the IAT of the packets received from the member nodes and uses the EWMA algorithm to detect a mean shift in event intensity during jamming attack. During jamming attack, this attack will be detected and the node generating the attack located and removed from the network. Alternatively, during the period of non-jamming attack, the packets in the cluster head will be processed and sent to the base station. In situation where the jamming attack is directed towards the cluster head, the base station receives the aggregated data from the cluster head and the detector installed on based station calculates the IAT of the received packet to detect a jamming attack using EWMA. If detected, the node generating the attack will be traced and removed from the network while legitimate packets will be stored if otherwise. The base station, in our work is located in a secure location close to the end user.

In summary, our jamming detection technique consists of two phases; the first phase is the training phase that involves the capture of normal IAT from legitimate member nodes to the cluster head and also from the cluster heads to the base station to initialize its parameters and obtain a normal profile. Just like in the work of Chabchoub et al. [34], no change point detection process will be performed during this phase. In the second phase, the test phase, a pattern change is detected during a jamming attack on a per packet basis using the EWMA algorithm. If an attack is detected, an alarm is triggered and the malicious node is removed from the network.

## 6. Simulation and Results

In this section, we simulate the jamming attack, a physical and MAC layer DoS attack that emits electromagnetic radiation into the communication channel to reduce the effective use of spectrum for legitimate transmission. The consequence of this is the indiscriminate consumption of resources and draining of the battery life of legitimate sensor nodes in WSN. For our simulation, we use the Community Resource for Archiving Wireless Data at Dartmouth “CRAWDAD” jamming attack dataset [36], which is made up of different jamming attacks obtained from a rural area located around Aachen in Germany. The CRAWDAD dataset is made up of constant jammers that vary in distance and attenuation, periodic jammers with varying attenuation, reactive jammers and cases of no jamming. The dataset consists of attributes such as node ID, source node ID, distance, time, packet size, transmit power, and received signal strength indicator.

The behavioural pattern of jamming attack can be determined by studying the IAT attribute of packets in a traffic flow. The cluster head monitors the member nodes in its cluster. To achieve this, the member nodes send information sensed to the cluster head. The cluster head in turn measures the IAT of the packet received before profiling a normal pattern during the non-jamming attack period. In a similar vein, the cluster heads send aggregated data to the base station and the base station measures the packet IAT metric to generate a normal profile during the non-jamming period. During the jamming attack period, our proposed EWMA algorithm is used to detect the jamming attacks in WSNs. We conduct experiments using packets of the non-jamming profile and jamming profiles for three different jamming attacks, namely; constant jamming, periodic jamming and reactive jamming. To analyse our work, we use the analyse-it tool [37].

**Figure 3:**
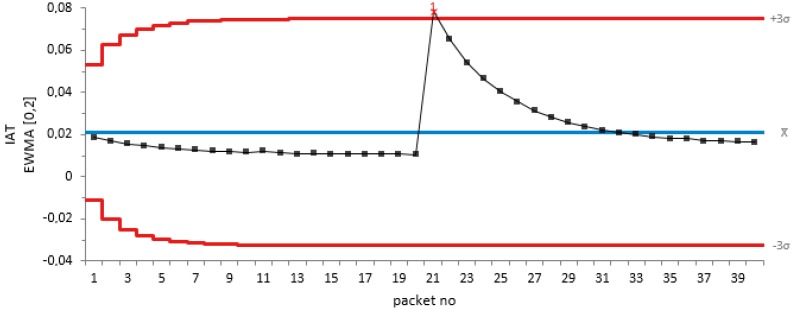
EWMA plot of the IAT metric against packet number for constant jamming attack.

For our trace simulation for constant jamming, we have used a jammer located 30 m from the target node to test the efficiency of our proposed algorithm. Table 1 shows the values of the out-of-control signals, process parameter and the process control statistics. Figure 3 shows the EWMA plot of the IAT metric against the packet number using 20 non-jamming packets and 20 packets of constant jamming attacks.

From Figure 3, it can be observed that our proposed method detects the jamming attack on the 21st packet, which is the start point of our simulated constant jamming attack traffic.

**Figure 4:**
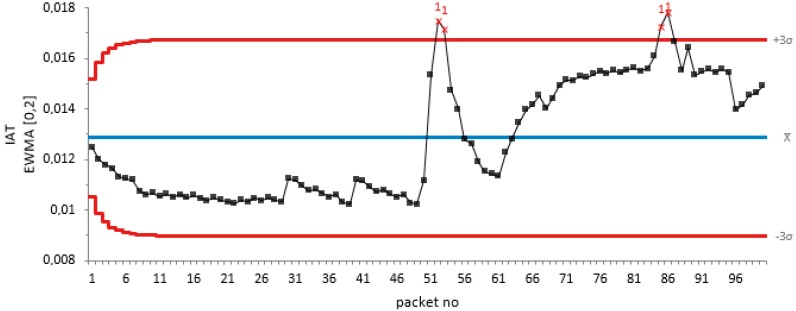
EWMA plot of the IAT metric against packet number for periodic jamming attack.

Similarly, for a periodic jamming attack at 20 dB attenuation for 50 non-jamming attack packets and 50 periodic jamming attacks, we test the efficiency of our proposed algorithm as shown in Figure 4.

From the graph of EMWA of the packet IAT against the packet number in Figure 4, it can be seen that there was a drift in the IAT sequence during the start of the jamming attack on the 51st packet. This shows that our proposed algorithm detects the change, thus tracking the periodic jamming attack.

**Figure 5:**
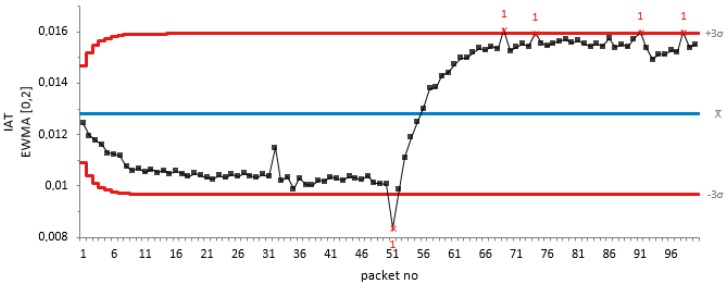
EWMA plot of the IAT metric against packet number for reactive jamming attack.

For reactive jamming attack, we use 50 packets for the non-jamming attacks and 50 packets for reactive jamming. Our algorithm has detected a shift in the traffic pattern during the jamming attack on packet number 51 which is the starting point of our simulated reactive jamming attack as shown in Figure 5 of the EWMA plot for IAT against packet number. The shift detected exceeded the out-off-control signal by first breaking the LCL before rising and braking the UPL. This again shows the efficiency of our proposed algorithm.

## 7. Discussion

To detect a jamming attack in a WSN, it is important to use a minimum number of metrics that can detect this attack to reduce complexity and associated overhead for resource-constrained sensor nodes. Therefore, in this work, we have proposed only the packet IAT to detect the change in traffic pattern during jamming attack using EWMA algorithm and deployed in the cluster head and base station with higher resources. Furthermore, the choice of packets IAT as compared to others makes it difficult for the attacker to manipulate our selected metric and still evade detection.

In evaluating our proposed algorithm, we have used a publicly available dataset, CRAWDAD [36] comprising of three different jamming attacks, namely constant jamming, periodic jamming and reactive jamming; together with a trace of no jamming. Our evaluations show that even with 20 packets of non-jamming and jamming trace, our proposed algorithm detects an abrupt change, thus showing the efficiency of our algorithm. Table 1 presents the result of our findings.

Our proposed technique has also been compared with other closely related proposed methods in the literature by considering the detection metric, jamming attack detected, simulator used and accuracy (see Table 2). We observe that most of the proposed methods produced a very high detection rate, just like our technique. However, most of these methods combined different detection techniques and use more than one detection metric, unlike our proposed technique, which tends to incur more overhead to the already resource constrained sensor node.

## 8. Conclusions

In this paper, we proposed a stepwise approach for detecting different forms of jamming attacks, where the jamming detector algorithm is deployed on the cluster head to detect attacks in the member nodes and also on the base stations to detect attacks in the cluster heads using the packet IAT metric. This metric is used to detect an abrupt change in packet sequence caused by a situation of jamming attacks using the EMWA algorithm. To demonstrate the efficiency of our proposed method, we conducted a trace-driven experiment using EWMA to detect changes in traffic flow during situations of both non-jamming and jamming attacks. We evaluated our work using a non-jamming trace and the three different variations of jamming attacks from the CRAWDAD dataset. Results obtained show that our proposed method can efficiently detect the presence of a jamming attack with little or no overhead in WSN.

Our detection approach can be implemented on sensor nodes deployed in areas such as military battle fields, healthcare and mission-critical events, where sensed information needs to be transmitted in real time devoid of error. In the future, we plan to extend the evaluation of our proposed method using other datasets and deploy in real-world environment.

## Figures and Tables

**Table 1:** Detection parameters for different jamming attacks.

Jamming Attacks	*n*		*λ*	Mean	*σ*	Detection Point (pck no)
	Jamming	Non-Jamming				
Constant	20	20	0.2	0.0538	0.021	21st
Periodic	50	50	0.2	0.0039	0.0129	51st
Reactive	50	50	0.2	0.00313	0.0128	51st

**Table 2:** Comparison of jamming detection approaches in WSN.

Approach	Detection Metrics	Jamming Attack Detected	Simulator	Accuracy
Error sample acquisition, interference detection and sequential jamming test [16]	RSS	Reactive	COTS BTnodes and Tmote Sky nodes	For ≥16 jammed bits: 100%
Query-based jamming detection algorithm (QUJDA) [23]	PDR, BPR and ECA	Reactive, Random, Constant, Cluster, Deceptive, Listen and Control	OMNET++	97% and above for varying jamming attacks.
Non-parametric cumulative sum (CUSUM) and weak estimation learning automata (WELA)-based scheme [26]	Bad Partial-Packet Ratio, Partial Packet-RSS and Deviation of PPRSS	Reactive	Aqua-sim	High
Artificial Bee Colony [38]	PDR, Energy, Distance, Packet Loss and RSS	Different Jamming attacks	MATLAB	High
Anomaly based Jamming Detection Algorithm (AJDA) [39]	PDR, BPR and ECA	Reactive, Random, Constant and Deceptive	OMNET++	≥98.75% for varying jamming attacks
Our method (EWMA)	IAT	Reactive, Constant and Periodic	Trace-driven	For ≥20 jammed packets: 100%
